# Recovery in soil carbon stocks but reduced carbon stabilization after near-natural restoration in degraded alpine meadows

**DOI:** 10.1038/s41598-024-82434-3

**Published:** 2024-12-28

**Authors:** Guoxing He, Xiaoni Liu, Yali Li, Heguang Xu, Tong Ji, Zhuoli Yang, Hao Qi, Chenglong Ma, Yunjun Wang, Degang Zhang, Dong Lin, Yafei Shi, Jiachang Jiang

**Affiliations:** 1 College of Grassland Science, Gansu Agricultural University, No. 1, Yingmen Village, Anning District, Lanzhou, 730070 Gansu China; 2 Key Laboratory of Grassland Ecosystem, Ministry of Education, Gansu Agricultural University, Lanzhou, 730070 Gansu China; 3 Sino-U.S. Centers for Grazing Land Ecosystem Sustainability, Lanzhou, 730070 Gansu China; 4 Pratacultural Engineering Laboratory of Gansu Province, Lanzhou, 730070 Gansu China; 5 Grassland Technique Extension Station of Gansu Province, Lanzhou, 730000 Gansu China

**Keywords:** Tibetan Plateau, Alpine meadow, Degradation, Near-natural restoration measures, Soil organic carbon fractions, Soil carbon stability, Grassland ecology, Restoration ecology, Environmental sciences

## Abstract

Near-natural restoration is acknowledged as an effective strategy for enhancing soil organic carbon (SOC) sequestration in degraded grasslands. However, the alterations in SOC fractions, stability, and relative sequestration capacity after restoration of degraded alpine meadows remain uncertain. In this study, we utilized the degraded alpine meadows on the northeastern edge of the Tibetan Plateau as a research area, with grazing as the control (CK) and restoration of 20 years of banned grazing (BG) and growing season resting grazing (RG). We analyzed the characteristics of SOC, SOC fractions, recalcitrant index (RI), and relative capacity of soil C sequestration (SCS_capacity_) under near-natural restoration measures. The objective of this study was to assess the recovery of SOC following near-natural restoration. The results showed that soil water content (SWC), SOC, soil total nitrogen (TN), and soil total phosphorus (TP) increased, while bulk density (BD) decreased in the degraded alpine meadow after near-natural restoration. In addition, near-natural restoration led to significant increases in particulate organic carbon (POC), readily oxidizable carbon (ROC), dissolved organic carbon (DOC), and microbial biomass carbon (MBC) content (*P* < 0.05). The SOC stock significantly increased, while the RI decreased. Compared to RG, BG had a greater increase in SOC stock. The study showed that 20 years of near-natural restoration in degraded alpine meadows mainly enhanced soil active carbon pools, while short-term restoration did not increase soil carbon stability. Therefore, avoiding re-exposure to overgrazing is essential to maintaining the restoration effect.

## Introduction

Soil serves as a critical carbon pool in terrestrial ecosystems, holding more soil organic carbon (SOC) than both vegetation and the atmosphere combined^1,2^. A little reduction in soil carbon may substantially impact atmospheric carbon dioxide (CO_2_) levels, significantly influencing the Earth’s climate system^3^. Consequently, the restoration of soil organic carbon stocks has become an important topic of current research, especially in degraded ecosystems. Grasslands are the largest terrestrial ecosystem in the world, accounting for about 40% of the land area^4^. The global grassland contributes about 34% to the soil organic carbon pools^5^, playing an important role in soil carbon cycle and carbon sequestration^4^.

However, approximately 49% of the world’s grasslands have experienced varying degrees of degradation due to the combined pressures of global warming and overgrazing in recent decades^6,7^. Grassland degradation accelerates SOC depletion, especially in alpine meadow ecosystems on the Tibetan Plateau^8^. The ecosystem exhibits considerable sensitivity and vulnerability due to its unique natural factors of high altitude, low temperatures, and intense radiation^9^. Alpine meadows are an important grassland type on the Tibetan Plateau, playing a crucial role in enhancing carbon sequestration and carbon sink. But its degradation has become increasingly serious due to fragile ecosystems, homogenous vegetation structure, and overgrazing^10^.

Sustained and high-intensity grazing activities in alpine meadows have limited plant regeneration and recovery, resulting in a decrease in vegetation coverage, aboveground biomass (AGB), belowground biomass (BGB), and accumulation of litter^11^. These factors reduce the ability of vegetation to protect the topsoil, leading to increased soil exposure, massive decomposition of soil organic matter, and ultimately the loss of soil organic carbon^12^. Moreover, local herders frequently utilize animal excrement as fuel, so diminishing the return of carbon to the soil and transforming the soil carbon pools from a “sink” to a “source”^13,14^. Therefore, the restoration of degraded grassland SOC is crucial for mitigating global climate change and a necessary safeguard for the sustainable development of grasslands.

In response to grassland degradation, scientists have proposed the concept of “near-natural restoration”^15^. This methodology emphasizes the “nature-based, return-to-nature” theory, utilizing the self-regulating properties of natural ecosystems to promote the restoration of degraded grasslands^16,17^. Banning grazing (BG) and resting grazing during the growing season (RG) are two commonly practiced near-natural restoration measures^8,18^. The main objectives of these measures are to reduce disturbance, enhance grassland quality, improve soil physicochemical properties, thereby mitigating the depletion of soil carbon pools and increasing the capacity of SOC sequestration in degraded grasslands^12^. Unlike BG, RG is closed to grazing throughout the growing season. Livestock forage on plants that have completed their life history, which protects the growing environment of the grassland^18^.

Currently, scholars use the recalcitrant index (RI) and the relative capacity of soil C sequestration (SCS_capacity_) when assessing the effects of ecological restoration measures on SOC fractions and soil carbon stability^19–21^. The RI could effectively reflect the effects of ecological restoration measures on soil carbon stability, while SCS_capacity_ could assess the relative sequestration capacity of SOC^21^. However, current research on grassland restoration measures mainly focused on SOC content^8,9,22^, and lacked research on the effects of near-natural restoration on SOC fractions and soil carbon stability in degraded alpine meadows. This study selected alpine meadows that had undergone near-natural restoration for 20 years on the northeastern edge of the Tibetan Plateau as research objective, aiming to explore the impact of near-natural restoration measures on SOC fractions and stability. We hypothesized that improvements in vegetation productivity and soil physicochemical properties following near-natural restoration would lead to an increase in the active SOC fractions, thereby increasing SOC stock, but soil carbon stability may not necessarily be improved. The increase in active SOC fractions due to near-natural restoration may lead to a decrease in soil carbon stability. We further utilized the soil active and recalcitrant carbon fractions to calculate the RI and SCS_capacity_, which were used to quantitatively assess the relative capacity of soil carbon sequestration after near-natural restoration.

## Results

### Variations of vegetation characteristics

**Fig. 1:**
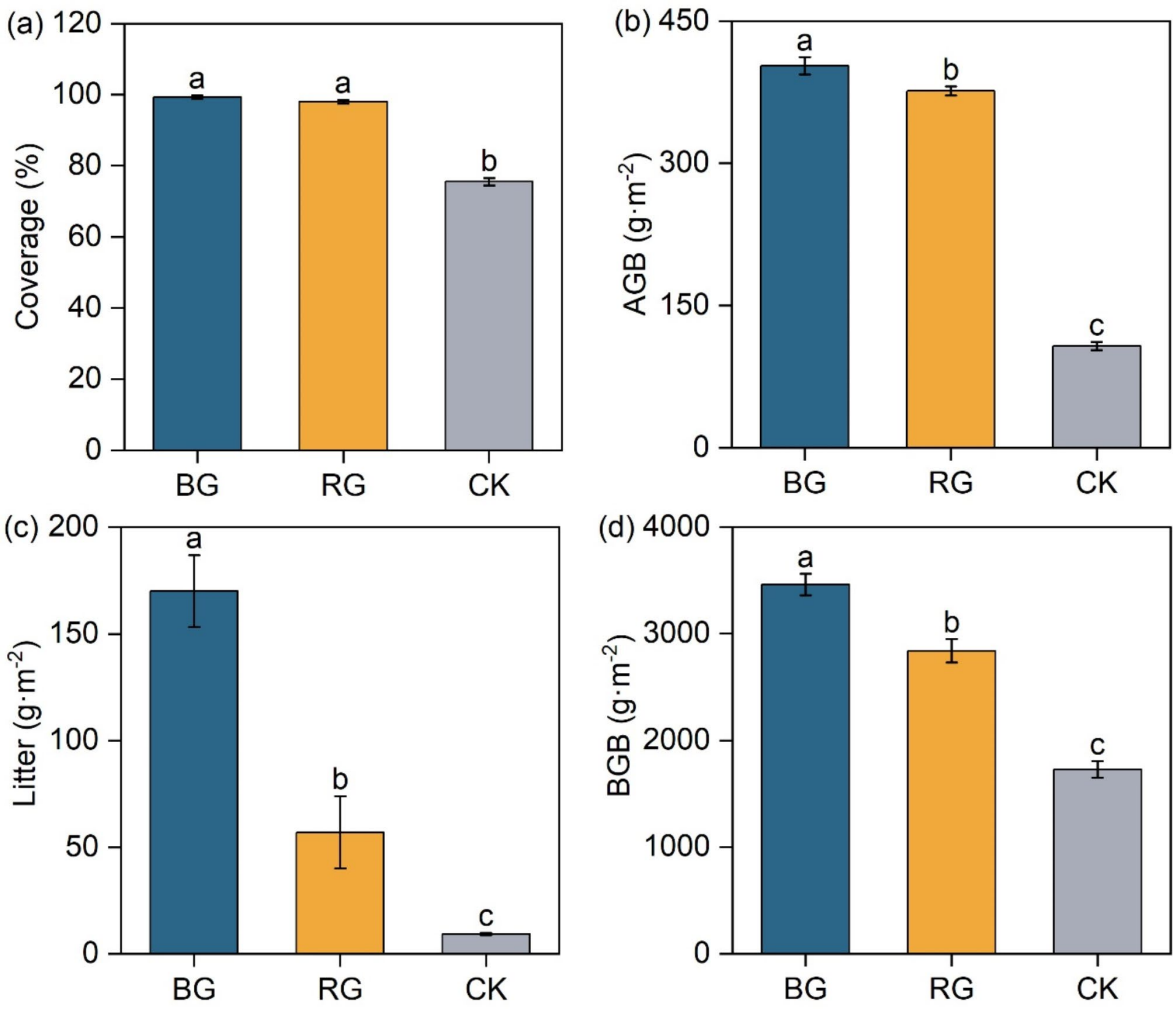
Variations of vegetation characteristics under different restoration measures. *Note*: Different lowercase letters indicate significant differences between the three restoration measures (*P* < 0.05, n = 9). *Coverage* vegetation total coverage; *AGB* aboveground biomass, *Litter* litter biomass, *BGB* belowground biomass.

The coverage of BG and RG increased by 30% and 29% compared to CK, respectively (*P* < 0.05) (Fig. 1a). The AGB, BGB, and Litter of BG and RG significantly increased compared to CK (*P* < 0.05) (Fig. 1b–d).

### Variations of soil properties

Overall, both restoration measures significantly altered soil physicochemical properties (Table 1).

In the topsoil, the SWC, TN, TP, and C:N were significantly higher in the BG than the CK. Between the two restoration measures, the soil TN content and C:P were significantly higher in the BG compared to the RG.

The physicochemical properties of the subsoil were less affected by the restoration measures compared to the topsoil (Table 1). The SWC and soil TP contents in the BG and RG were significantly higher than those in the CK. (*P* < 0.05) (Table 1). The BD of BG was significantly lower compared to CK (*P* < 0.05) (Table 1).

**Table 1:** Variations of soil physicochemical properties.

Depth (cm)	Measures	SWC (%)	BD (g cm^−3^)	TN (g kg^−1^)	TP (g kg^−1^)	C:N	C:P	N:P
0–20	BG	26.9 ± 0.5^a^	0.75 ± 0.05^b^	6.75 ± 0.21^a^	0.79 ± 0.04^a^	13.02 ± 0.75^a^	111.59 ± 6.81^a^	8.63 ± 0.38^ab^
	RG	23.9 ± 0.5^b^	0.78 ± 0.01^b^	6.07 ± 0.12^b^	0.82 ± 0.05^a^	12.21 ± 0.23^ab^	92.14 ± 5.37^b^	7.54 ± 0.40^b^
	CK	18.2 ± 0.8^c^	1.04 ± 0.03^a^	5.79 ± 0.10^b^	0.65 ± 0.03^b^	10.81 ± 0.30^b^	97.88 ± 5.18^ab^	9.05 ± 0.41^a^
20–40	BG	22.1 ± 0.4^a^	0.89 ± 0.03^b^	6.12 ± 0.19^a^	0.74 ± 0.06^a^	10.81 ± 0.38^a^	93.90 ± 7.81^a^	8.79 ± 0.81^a^
	RG	21.6 ± 0.5^a^	0.98 ± 0.02^a^	5.40 ± 0.14^b^	0.62 ± 0.04^a^	11.85 ± 0.54^a^	104.27 ± 5.73^a^	8.97 ± 0.70^a^
	CK	18.0 ± 1.0^b^	1.01 ± 0.02^a^	5.26 ± 0.17^b^	0.59 ± 0.02^b^	11.58 ± 0.48^a^	103.63 ± 4.73^a^	9.01 ± 0.42^a^

Note: Different lowercase letters indicate significant differences between the three repair methods (*P* < 0.05, n = 9).

*BG* banned grazing, *RG* rest grazing during the growing season, *CK* grazing, *SWC* soil water content, *BD* bulk density, *TN* total nitrogen, *TP* total phosphorus, *C: N* carbon-to-nitrogen ratio, *C:P* carbon-to-phosphorus ratio, *N:P* nitrogen-to-phosphorus ratio.

### Variations of SOC content and SOC stock

**Fig. 2:**
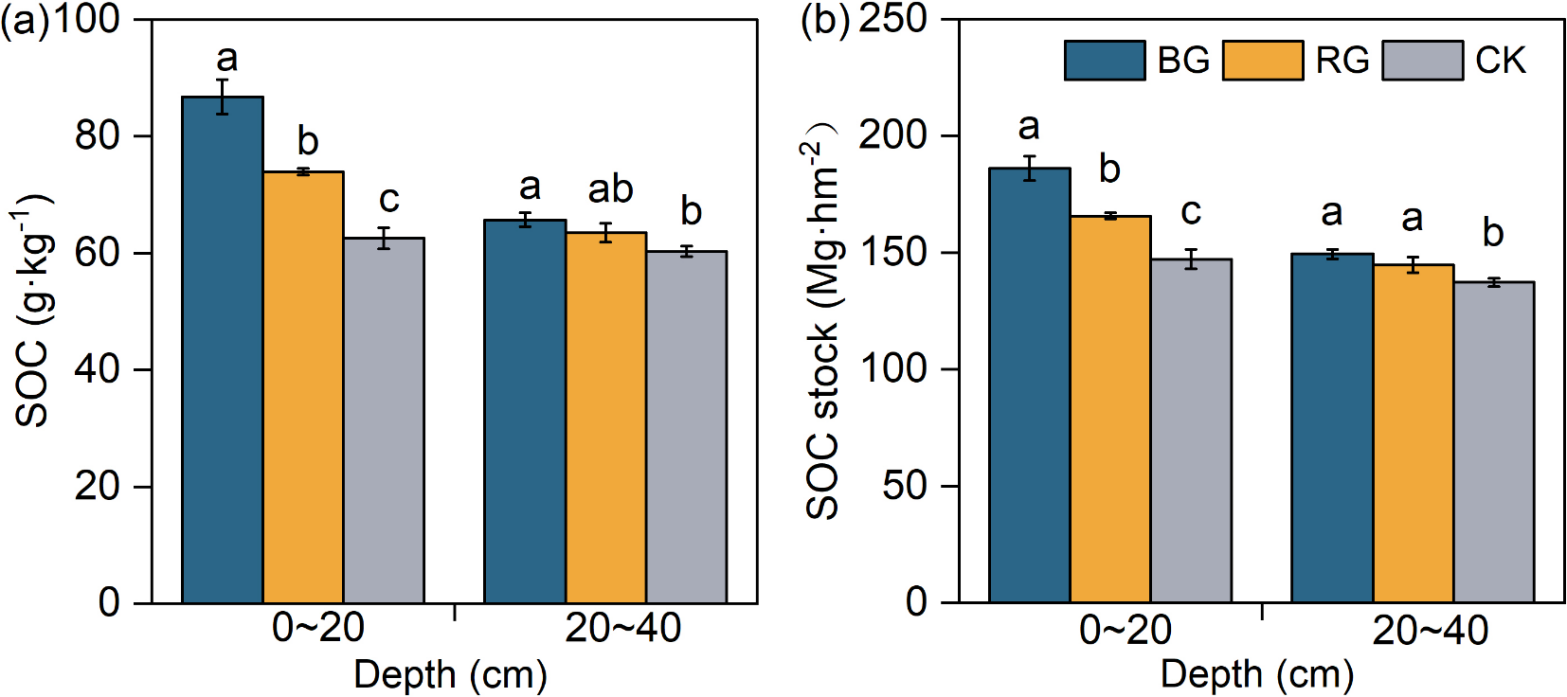
Comparison of SOC and SOC stock under different restoration measures. *Note*: Different lowercase letters indicate significant differences (*P* < 0.05, n = 9); *SOC* soil organic carbon, *SOC stock* soil organic carbon stock.

In the topsoil, the SOC content significantly increased by 39% and 18% in BG and RG, respectively, compared to CK (*P* < 0.05) (Fig. 2a). In the subsoil, the SOC content significantly increased by 9% under BG compared to CK (*P* < 0.05) (Fig. 2a).

The two restoration measures significantly increased SOC stock (*P* < 0.05) (Fig. 2b). In the topsoil, SOC stock significantly increased by 26% and 13% in BG and RG (*P* < 0.05), respectively, compared to CK (Fig. 2b). In the subsoil, the SOC stock of BG and RG significantly increased by 9% and 5% (*P* < 0.05), respectively, compared to CK (Fig. 2b).

### SOC fractions and RI

**Fig. 3:**
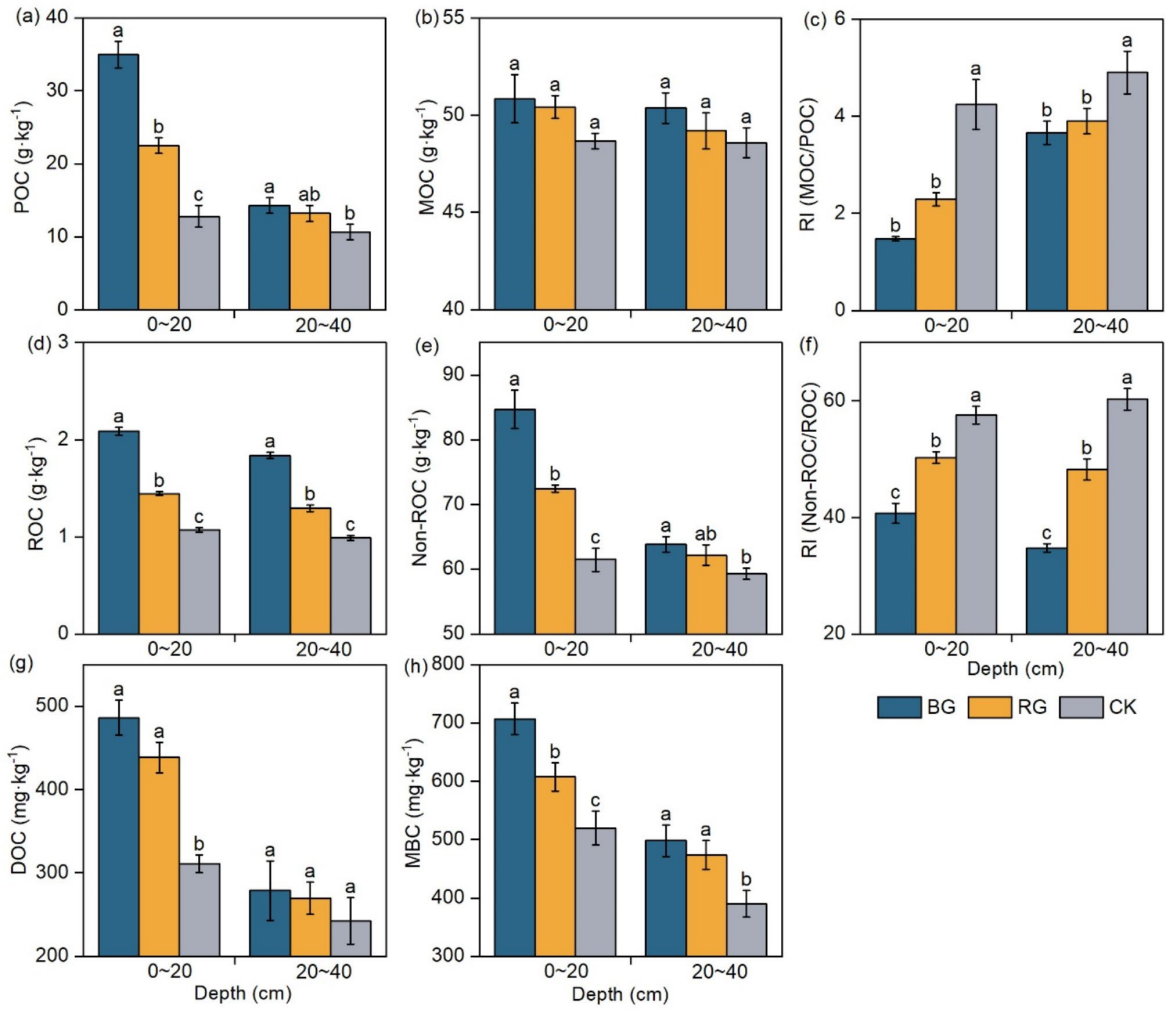
Comparison of SOC fractions under different restoration measures. *Note*: Different lowercase letters indicate significant differences (*P* < 0.05, n = 9); *POC* particulate organic carbon, *MOC* mineral-associated organic carbon, *ROC* readily oxidizable carbon, *Non-ROC* non-readily oxidizable carbon, *RI* POC/MOC or Non-ROC/ROC, *DOC* dissolved organic carbon, *MBC* microbial biomass carbon.

In the physical classification of the SOC fractions, the POC content showed BG > RG > CK in the topsoil (Fig. 3a). In the subsoil, the POC content was significantly higher in the BG than the CK (*P* < 0.05). But there was no significant difference between the two restoration measures in the subsoil (Fig. 3a). Both the two restoration measures showed no significant difference in MOC content compared to the CK (*P* > 0.05) (Fig. 3b). Furthermore, the physical RI values exhibited an inverse relationship with POC content. The physical RI significantly decreased after near-natural restoration, with significant differences between BG, RG, and CK (*P* < 0.05) (Fig. 3c).

In the chemistry classification of the SOC fractions, the soil ROC content was significantly increased for BG and RG compared to CK (*P* < 0.05) (Fig. 3d). The Non-ROC content showed significant differences between BG and CK (*P* < 0.05) (Fig. 3e). The chemical RI values exhibited similarities to the physical RI values. The BG and RG were significantly lower compared to the CK (*P* < 0.05) (Fig. 3f).

In the topsoil, the DOC content of BG and RG increased significantly by 56% and 41%, compared to CK, respectively. There was no significant difference between the DOC content in the subsoil (Fig. 3g).

The soil MBC content was significantly increased in the BG and RG (*P* < 0.05) (Fig. 3h).

The proportions of MOC/SOC and Non-ROC/SOC were 58.5-80.7% and 97.2-98.4%, respectively. This indicated that MOC and Non-ROC collectively constituted a significant proportion of the SOC (Table 2). In the topsoil, the proportions of POC/SOC and ROC/SOC both BG and RG were significantly higher than CK (*P* < 0.05). Conversely, the proportions of MOC/SOC and Non-ROC/SOC were significantly lower than in the CK (*P* < 0.05). Additionally, the proportions of DOC/SOC in the RG were significantly higher than CK (*P* < 0.05) (Table 2). In the subsoil, the proportions of POC/SOC, ROC/SOC, and MBC/SOC in the BG were all significantly higher than CK (*P* < 0.05) (Table 2).

**Table 2:** The proportions of SOC fractions accounting for SOC in different restoration measures.

Depth (cm)	Measures	POC/SOC (%)	MOC/SOC (%)	ROC/SOC (%)	Non-ROC/SOC (%)	DOC/SOC (× 10^–4^)	MBC/SOC (× 10^–4^)
0–20	BG	40.07 ± 0.81^a^	58.75 ± 0.80^c^	2.43 ± 0.11^a^	97.57 ± 0.11^c^	56.78 ± 3.61^ab^	81.35 ± 0.75^a^
	RG	30.36 ± 1.19^b^	68.26 ± 1.19^b^	1.96 ± 0.04^b^	98.04 ± 0.03^b^	59.40 ± 2.67^a^	82.31 ± 3.63^a^
	CK	20.06 ± 1.73^c^	78.16 ± 1.69^a^	1.72 ± 0.04^c^	98.28 ± 0.04^a^	50.03 ± 2.31^b^	83.45 ± 4.93^a^
20–40	BG	21.64 ± 1.29^a^	76.73 ± 1.23^b^	2.80 ± 0.05^a^	97.20 ± 0.05^c^	42.61 ± 5.48^a^	75.83 ± 3.94^a^
	RG	20.60 ± 1.31^ab^	77.71 ± 1.28^ab^	2.06 ± 0.08^b^	97.94 ± 0.08^b^	42.51 ± 2.71^a^	74.66 ± 3.41^ab^
	CK	17.51 ± 1.54^b^	80.67 ± 1.53^a^	1.64 ± 0.05^c^	98.35 ± 0.05^a^	40.17 ± 4.53^a^	64.60 ± 3.62^b^

Note: Different lowercase letters indicate significant differences (*P* < 0.05).

*SOC* soil organic carbon, *MOC* mineral-associated organic carbon, *POC* particulate organic carbon, *ROC* readily oxidizable carbon, *Non-ROC* non-readily oxidizable carbon, *DOC* dissolved organic carbon, *MBC* microbial biomass carbon.

### Relative capacity of soil C sequestration

The implementation of the two restoration measures led to a significant increase in SOC_stock_ (*P* < 0.05), whereas there was no significant increase in SCS_capacity_ (Table 3).

In topsoil, SCS_stock_ of BG and RG was significantly higher than CK (*P* < 0.05) (Table 3). This indicated that the relative accumulation of SOC significantly increased following the implementation of the two restoration measures (*P* < 0.05). The SCS_RI_ showed a consistent pattern in the physical and chemical groups. The SCS_RI_ values under BG and RG exhibited significantly lower compared to CK (*P* < 0.05), as shown in Table 3. Based on the SCS_capacity_ of physical components, BG and RG were significantly lower than CK (*P* < 0.05). There was no significant difference (*P* > 0.05) between BG, RG, and CK based on SCS_capacity_ of chemical components (Table 3).

In subsoil, the SCS_capacity_ based on physical components was not significantly different between BG, RG, and CK, while the SCS_capacity_ based on chemical components was significantly lower for BG and RG than CK (*P* < 0.05) (Table 3).

**Table 3:** Comparative analysis of SOC fraction sequestration capacity based on physical and chemical methods under different restoration measures.

Depth (cm)	Measures	SCS_stock_	Physical analyses		Chemistry analyses	
			SCS_RI_	SCS_capacity_	SCS_RI_	SCS_capacity_
0–20	BG	1.40 ± 0.06^a^	0.39 ± 0.05^c^	0.52 ± 0.05^c^	0.71 ± 0.04^c^	1.01 ± 0.09^a^
	RG	1.19 ± 0.04^b^	0.61 ± 0.08^b^	0.70 ± 0.08^b^	0.88 ± 0.03^b^	1.06 ± 0.07^a^
	CK	1^c^	1^a^	1^a^	1^a^	1^a^
20–40	BG	1.09 ± 0.02^a^	0.81 ± 0.11^a^	0.88 ± 0.11^a^	0.58 ± 0.02^c^	0.64 ± 0.03^c^
	RG	1.05 ± 0.03^ab^	0.85 ± 0.11^a^	0.87 ± 0.08^a^	0.80 ± 0.04^b^	0.85 ± 0.05^b^
	CK	1^b^	1^a^	1^a^	1^a^	1^a^

Note: Different lowercase letters indicate significant differences (*P* < 0.05).

*SCS*_*stock*_ SOC of restoration/SOC of CK, *SCS*_*RI*_ RI of restoration/RI of CK, *SCS*_*capacity*_ relative capacity of soil C sequestration.

## Discussion

### Effects of near-natural restoration measures on SOC content and stocks

In this study, we found that the SOC content increased significantly following near-natural restoration in degraded alpine meadows. It suggests that BG and RG measures are favorable to the soil carbon sink enhancement in alpine meadows. At the same time, we observed that the BG and RG measures significantly increased vegetation coverage compared to CK (Fig. 1a). Increased vegetation coverage effectively reduces the bare area of soil, thereby protecting the soil from wind and water erosion. This protective effect prevents the loss of SOC in grassland^23^.

On the other hand, both BG and RG measures significantly increased AGB (Fig. 1b). The increase in AGB contributes to the formation of the litter layer (Fig. 1c), which serves as a rich source of organic matter input for the soil. BG and RG measures also promoted the formation of well-developed plant root systems (Fig. 1d), resulting in greater production of root residue material. The surface litter and plant root residues are gradually decomposed by soil microorganisms and protozoa^24^. During this decomposition process, carbon from plant residue is released into the soil, and this released carbon serves as an important source of SOC^24–26^.

In addition, BG and RG measures significantly reduced soil BD (Table 1). Previous studies have shown that soils with lower BD can store more organic matter^27,28^. Since organic matter can move through soil pores, soils exhibiting lower BD usually have more macro-pores, thereby increasing the contact area between soil microorganisms and organic matter. This allowed more organic matter to decompose, thereby increasing SOC contents^28^. Consequently, near-natural restoration measures reduced the amount of soil carbon loss and increased carbon input in degraded alpine meadows. These are the main reasons for the increase in SOC content.

The study showed that banned grazing had a significant effect on increasing SOC stocks in degraded grassland^22,29,30^. This is consistent with the results of this study. The SOC stock significantly increased after the implementation of near-natural restoration measures in degraded alpine meadows (Fig. 2).

### Effects of near-natural restoration measures on SOC fractions and stability

In this study, BG and RG measures significantly increased soil POC content (Fig. 3a), but they did not lead to an increase in the more stable MOC content (Fig. 3b). BG was more effective in enhancing soil POC content compared to RG (Fig. 3a). The POC is often defined as an important indicator of the soil active carbon pools and is sensitive to changes in soil quality. Thus it is also more responsive to restoration measures^9^. After near-natural restoration, plant litter is decomposed and enters the soil as POC, which is an important source of SOC^3,25,26^. The MOC has excellent stability because it is protected by physical and chemical interactions. Microbial breakdown and utilization for MOC is difficult, and consequently, its persistence usually lasts for decades to centuries^31^. Hence, the MOC is less responsive to near-natural restoration measures. The pattern of soil ROC content was similar to that of POC (Fig. 3d). The above results showed that near-natural restoration measures mainly increased the active carbon pools of the soil, and BG was more effective compared to RG in degraded alpine meadows.

Notably, the increased soil carbon pools after near-natural restoration, particularly in the active carbon pools, do not necessarily imply an improvement in the long-term sequestration capacity and stability of soil carbon pools^32^. The stability of soil carbon pools is directly related to the quality and sustainability of soil carbon sequestration, which is an important reflection of the function of soil carbon sinks. The RI and SCS_capacity_ are usually used to assess the sensitivity of SOC sequestration^21,32^. They are important for assessing soil carbon pools after near-natural restoration^21^. Specifically, following the restoration of alpine meadows, a significant increase in SOC stocks was observed (Fig. 2b), whereas both physical RI and chemical RI decreased significantly (Fig. 3c,f). This was associated with a significant increase in the active carbon pool, whereas the recalcitrant carbon pool remained stable following near natural restoration of degraded alpine meadows. This was attributed to the fact that BG and RG measures facilitate sustained vegetation recovery such as significant increases in AGB, BGB, and Litter biomass and changes in soil structure. Consequently, plant residues that input the soil are more readily decomposed, and the resultant decomposed organic matter accumulates as POC, thereby leading to a decrease in RI values^25^. In addition, the active carbon fraction always recovers in a short period of time, but the recovery of the stabilized carbon fraction takes several decades. And plant roots may grow rapidly to take up more nutrients from the active organic carbon fraction^32^. This indicated that near-natural restoration measures significantly increased the active carbon fractions (POC and ROC), but the inert carbon (MOC and Non-ROC) remained stable in the degraded alpine meadow. As a result, RI decreased significantly and SCS_capacity_ remained constant or decreased (Table 2).

In conclusion, the soil active carbon fraction increased significantly and RI decreased significantly after the near-natural restoration of degraded alpine meadows. This indicated that these measures can effectively enhance the SOC content, but the soil carbon stability did not increase. When alpine meadows are disturbed again, this may result in the loss of significant active carbon pools. This is because it has a low stabilizing capacity of the soil carbon pools. Therefore, it is particularly important to avoid overgrazing after restoration. Meanwhile, it was found that BG was more favorable to enhance soil active carbon pools than RG in degraded alpine meadows. The relative sequestration capacity of soil carbon had not been enhanced by either BG or RG. Thus, its carbon pool is relatively unstable. The restoration period in this study was relatively short, spanning only 20 years, and future studies need longer time scales to comprehensively assess the long-term stability characteristics and sequestration capacity of soil carbon pools following near-natural restoration of degraded alpine meadows.

## Conclusion

After 20 years of near-natural restoration of degraded alpine meadows, both BG and RG significantly increased SOC, POC and ROC contents, but MOC contents were not significantly increased. Meanwhile, the POC and ROC contents in the BG were significantly higher than those in the RG. On the other hand, the relative sequestration capacity of SOC has not improved due to the decline of soil RI. These results indicated that short-term restoration of degraded alpine meadows was mainly accompanied by the accumulation of soil active carbon pools, particularly those that were relatively unstable and vulnerable to disturbance over the short-term. Therefore, it is particularly important to avoid overgrazing again after the near-natural restoration of degraded alpine meadows.

## Materials and methods

### Study site

**Fig. 4:**
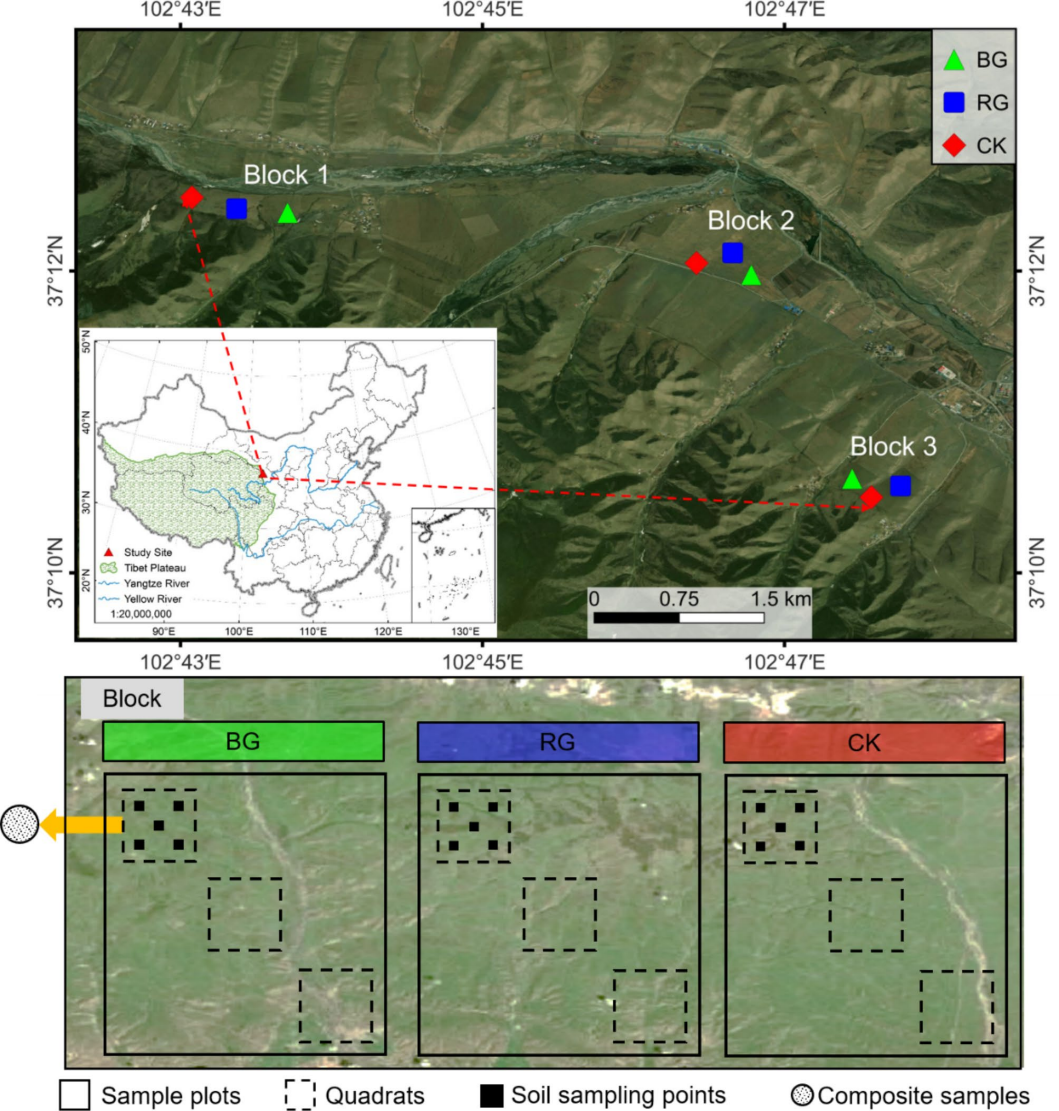
Spatial distribution of sampling sites. Note: BG: banned grazing; RG: rest grazing during the growing season; CK: grazing. *Statement. Based on the standard map supervised by the Ministry of Natural Resources of the People’s Republic of china [No. GS (2022) 4316] and [No. ZS(2022) 004] retrieved from: http://bzdt.ch.mnr.gov.cn/browse.html?picId=%224o28b0625501ad13015501ad2bfc0690%22 and http://zrzyt.xizang.gov.cn/fw/zyxz/202004/t20200430/139102.html. The map was created using Arc GIS 10.2 (https://www.esri.com/software/ArcGIS).

The study area is located in Wuwei City, Gansu Province, China (37° 11′ N, 102° 46′ E), and lies on the northeastern edge of the Tibetan Plateau (Fig. 4). The climate in the study area resembles that of the Tibetan Plateau, characterized by cold and humid conditions throughout most of the year, along with low air pressure, reduced oxygen levels, intense sunshine, and high levels of ultraviolet radiation. This region is characterized by a continental plateau monsoon climate, with mean annual temperature and precipitation of 0.16 °C and 416.9 mm, respectively. The majority of precipitation falls between July and September in the study area. The duration of the growing season ranges from 120 d to 140 d. The soil type is an alpine chernozem. The grasslands ecosystem is classified as an alpine meadow. The dominant vegetation comprises *Elymus nutans*, *Poa crymophila* and *Kobresia humilis*^33^. The animals primarily forage on the leaves and stems of herbaceous plants that have completed their growing cycle during the non-growing season.

### Experimental design and sample collection

The study site selected for analysis was a moderate-deteriorated alpine meadow^34^, which underwent the “Returning Grazing Land to Grassland” initiative in 2003. The site’s natural characteristics, including terrain, slope direction, and steepness, remained consistent throughout the project. The grazing animals present in the area were indigenous Tibetan sheep and yaks. Two types of near-natural restoration measures were implemented, specifically BG and RG. A randomized block experimental design was utilized, with grazed sample plots serving as the control (CK). Three blocks were established, each containing three treatments, resulting in a total of nine sample plots. Each sample plot covered an area of 100 m × 100 m. BG involved no grazing throughout the year, stocking rates: 0 sheep unit hm^−2^ a^−1^. RG entailed no grazing from the end of April to the end of September annually, stocking rates: 3.07 sheep unit hm^−2^ a^−1^. CK represented continuous grazing throughout the year, stocking rates: 6.13 sheep unit hm^−2^ a^−1^ (Fig. 4).

Plant community surveys and soil sampling were conducted in mid-August 2022, with three random quadrats (1 × 1 m) set up in each sampling plot, maintaining a minimum distance of 50 m between quadrats. A total of 27 quadrats were established (three blocks × three treatments × three quadrats per treatment). Vegetation coverage, AGB, BGB, and litter biomass (Litter) within each quadrat were surveyed. Vegetation coverage was measured using point-intercept sampling, employing a 1 m × 1 m square frame, with 100 sampling points spaced equidistantly within the frame. AGB was measured using the harvest method. Subsequently, within each harvested plot, a root auger (Φ = 90 mm) and a soil auger (Φ = 38 mm) were used to collect belowground biomass and soil samples, respectively, following the soil layers of 0–20 cm (topsoil) and 20–40 cm (subsoil). Soil samples were collected using the “five-point method”, where every five augers were combined to form one replicate. Three replicates were collected per plot, resulting in a total of 54 soil samples. Soil bulk density (BD) was determined using the “ring cutter method” during the soil sampling process. Roots were separated from the soil using the mesh bag method. All aboveground vegetation, litter, and root samples were killed green at 105 °C for 30 min, then dried to a constant weight in a 65 °C oven. After drying, the samples were weighed and the biomass was calculated. Soil samples were air-dried, roots and debris were removed, and the samples were sifted through a 2 mm mesh screen before analyzing the soil’s physicochemical properties and SOC fractions.

### Sample analysis

Soil water content (SWC) was determined by weighing 10 g of wet soil samples and subsequently drying them in an oven at 105 °C for 24 h. Soil bulk density (BD) was determined using the ring knife method with a volume of 100 cm^3^ to collect undisturbed soil samples. Subsequently, the samples were dried in an oven until reaching a constant weight, after which they were weighed^35^.

SOC content was assessed through the oxidation of concentrated sulfuric acid-potassium dichromate and subsequent titration with ferrous sulfate. The soil total nitrogen (TN) content was analyzed using an automatic carbon and nitrogen analyzer (Primacs SNC 100-IC-E; Sklar, Netherlands). The soil total phosphorus (TP) content was employed by the molybdenum-antimony-ascorbic acid colorimetric method^9^. Dissolved organic carbon (DOC) in the soil was extracted using a 0.5 mol L^−1^ potassium sulfate (K_2_SO_4_) solution with a soil-water ratio of 1:4. DOC was separated using a 0.45 μm filter membrane and quantified using an automatic carbon and nitrogen analyzer^36^. Microbial biomass carbon (MBC) was quantified using the chloroform fumigation extraction method^37^.

Soil particulate organic carbon (POC) and mineral-associated organic carbon (MOC) content were isolated using wet sieving^38–40^. 10 g of soil samples were weighed and extracted from a 2 mm mesh screen. The samples were dispersed using 30 mL of a 5 g L^−1^ solution of sodium hexametaphosphate ((NaPO_3_)_6_) and subjected to agitation in a reciprocating vibrator operating at 90 r min^−1^ for 6 h. Subsequently, the samples were further dispersed with deionized water. The fraction retained on the sieve and the fraction that passed through the sieve were subsequently transferred to a beaker for drying at 60 °C, followed by weighing and individual analysis of soil carbon content. Soil POC content (> 53 μm) and MOC content (< 53 μm) can be determined.

The concentration of readily oxidizable carbon (ROC) was determined through oxidation using 0.02 mol L^−1^ potassium manganate (KMnO_4_)^41^. Non-ROC, classified as a non-labile fraction of carbon, was determined by subtracting ROC from SOC.

### SOC sequestration capacity

Zhang et al.^21^ proposed the concept of SCS_capacity_, which can be expressed as follows:

1 $${\text{SCS}}_{{{\text{capacity}}}} = {\text{SCS}}_{{{\text{stock}}}} \times {\text{SCS}}_{{{\text{RI}}}}$$

where SCS_stock_ and SCS_RI_ represent the changes in soil carbon stock and its RI when comparing restored alpine meadow plots to those without restoration. SCS_stock_ and SCS_RI_ can be calculated as follows:

2 $${\text{SCS}}_{{{\text{stock}}}} = {\text{SOC of restoration}}/{\text{SOC of CK}}$$

3 $${\text{SCS}}_{{{\text{RI}}}} = {\text{RI of restoration}}/{\text{RI of CK}}$$

4 $${\text{RI}} = {\text{MOC}}/{\text{POC or Non-ROC}}/{\text{ROC}}$$

### SOC stock

The “equivalent soil mass” method is used to estimate SOC stock across different layers of a 40 cm soil profile, typically evaluating every 20 cm segment^42,43^.

5 $${M}_{soil, i}={BD}_{i}\times {T}_{i}\times 10000$$

6 $${SOC}_{stock}=\sum_{i=1}^{n}[{M}_{soil, i}\times {con}_{i}+({M}_{o,i}-{M}_{soil, i})\times {conc}_{i+1}]\times {A}_{i}\times 0.001$$

where *M*_*soil*, *i*_ is the soil mass (Mg ha^−1^) of the *i*th layer (*i* = 1, 2, representing the depths of 0–40 cm, respectively); BD_*i*_ and T_*i*_ are BD (g cm^−3^) and thickness (m) in the *i*th layer, respectively; conc_*i*_ and conc_*i*+1_ are the concentrations of SOC in the *i*th and *i* + 1th layers (g kg^−1^), respectively; and *M*_*o*, *i*_ is the maximum soil mass from the first layer to the *n*th layer.

### Statistical analysis

The homogeneity of variances in all data sets was assessed using the least significant difference (LSD) test available in the ‘car’ package^44^, followed by a normality test conducted using Shapiro-Wilk test^10^. The analyses were conducted utilizing R version 4.0.3^45^.

## Data Availability

Data available on reasonable request from corresponding author.
